# Avocado‐derived extracellular vesicles loaded with ginkgetin and berberine prevent inflammation and macrophage foam cell formation

**DOI:** 10.1111/jcmm.18177

**Published:** 2024-03-17

**Authors:** Shweta Sharma, Manisha Mahanty, Suneha G. Rahaman, Pritha Mukherjee, Bidisha Dutta, Mohammad Imran Khan, Karunakaran Reddy Sankaran, Xiaoming He, Lakshmyya Kesavalu, Wei Li, Shaik O. Rahaman

**Affiliations:** ^1^ Department of Nutrition and Food Science University of Maryland College Park Maryland USA; ^2^ Fischell Department of Bioengineering University of Maryland College Park Maryland USA; ^3^ Department of Periodontology and Oral Biology, College of Dentistry University of Florida Gainesville Florida USA; ^4^ Department of Biomedical Sciences, Joan C. Edwards School of Medicine Marshall University Huntington West Virginia USA

**Keywords:** avocado‐derived extracellular vesicles, macrophage foam cells, nutraceutical, oxLDL

## Abstract

Atherosclerosis, a chronic inflammatory disease of aorta, remains the major cause of morbidity and mortality among cardiovascular disease patients. Macrophage foam cell formation and inflammation are critically involved in early stages of atherosclerosis, hence chemopreventive targeting of foam cell formation by nutraceuticals may be a promising approach to curbing the progression of atherosclerosis. However, many nutraceuticals including berberine and ginkgetin have low stability, tissue/cell penetration and bioavailability resulting in inadequate chemotherapeutic effects of these nutraceuticals. We have used avocado‐derived extracellular vesicles (EV) isolated from avocado (EV^Avo^) as a novel carrier of nutraceuticals, in a strategy to alleviate the build‐up of macrophage foam cells and expression of inflammatory genes. Our key findings are: (i) Avocado is a natural source of plant‐derived EVs as shown by the results from transmission electron microscopy, dynamic light scattering and NanoBrook Omni analysis and atomic force microscopy; (ii) EV^Avo^ are taken up by macrophages, a critical cell type in atherosclerosis; (iii) EV^Avo^ can be loaded with high amounts of ginkgetin and berberine; (iv) ginkgetin plus berberine‐loaded EV^Avo^ (EV^Avo(B+G)^) suppress activation of NFκB and NLRP3, and inhibit expression of pro‐inflammatory and atherogenic genes, specifically *Cd36*, *Tnfα*, *Il1β* and *Il6*; (v) EV^Avo(B+G)^ attenuate oxidized low‐density lipoprotein (oxLDL)‐induced macrophage foam cell formation and (vi) EV^Avo(B+G)^ inhibit oxLDL uptake but not its cell surface binding during foam cell formation. Overall, our results suggest that using EV^Avo^ as a natural carrier of nutraceuticals may improve strategies to curb the progression of atherosclerosis by limiting inflammation and pro‐atherogenic responses.

## 1 INTRODUCTION

Cardiovascular disease (CVD) remains the major cause of morbidity and mortality globally, despite substantial scientific advancement in understanding the disease pathogenesis.^1^, ^2^ It has been reported that approximately one third of global deaths are linked to CVD‐related events.^3^ Given the surge in prevalence of obesity and diabetes, the worldwide occurrence of CVD is expected to rise and to inflict a greater public health and economic burden throughout the world. Atherosclerosis, a chronic arterial disease affecting large and medium arteries, is the primary contributor to CVD‐related events, including myocardial infarction and stroke.^1^, ^2^ During early atherogenesis, as a result of inflammation and endothelial dysfunction, blood monocytes transmigrate into the aortic intimal areas, where they differentiate into tissue macrophages.^4^, ^5^ In aortic intimal spaces, binding and internalization of oxidized low‐density lipoprotein (oxLDL) by macrophages aided by scavenger receptors like SRA and CD36 generates a feed‐forward athero‐inflammatory loop resulting in development of lipid‐laden foam cells, a critical early event in atherosclerosis.^4^, ^5^, ^6^, ^7^, ^8^, ^9^, ^10^, ^11^, ^12^ Cascades of athero‐inflammatory events provoked by macrophage foam cells lead to the development of an early fatty streak and eventually the appearance of advanced atherosclerotic plaque.^4^, ^5^, ^6^, ^7^, ^8^, ^9^, ^10^, ^11^, ^12^ Subsequently, the development of unstable plaque can trigger thrombosis and heart attack. Although currently approved pharmacotherapies for CVD are effective in reducing mortality, a large percentage of patients receiving current therapies still harbour a discernible residual risk of a CVD‐linked complication, and show unwanted side effects such as muscle pain and hepatic toxicity.^1^, ^2^, ^3^, ^13^, ^14^, ^15^, ^16^ There is an urgent need, therefore, to develop alternative, safe and targeted therapeutics for atherosclerosis that can be administered alone or in combination with current pharmacotherapies to limit inflammation and other pro‐atherogenic responses in atherosclerosis.

One potential chemopreventive avenue being explored for the prevention of atherosclerosis is natural product‐derived nutraceuticals that are reported to have anti‐inflammatory properties.^16^ Nutraceuticals are defined as ‘phytocomplex and the pool of secondary metabolites concentrated and administered in the proper pharmaceutical form to provide beneficial health effect’.^17^ As for an example, berberine, an alkaloid and ginkgetin, a biflavonoid, are known to have anti‐oxidative and anti‐inflammatory effects and have been shown to be beneficial against various inflammatory/oxidative conditions in vitro and in vivo.^18^, ^19^, ^20^, ^21^, ^22^, ^23^ It was reported that berberine treatment prevented oxLDL‐induced macrophage foam cell formation and high‐fat diet‐induced atherosclerosis in atherosclerosis‐prone hyperlipidemic ApoE‐null or LDLR‐null mice.^18^, ^19^, ^22^, ^23^ Similarly, Lian et al. showed a therapeutic benefit of ginkgetin in atherosclerotic rats.^24^ However, both berberine and ginkgetin have low stability, and because of their lipophobicity, penetration to target tissues/cells is also low. Pharmacokinetic studies have shown low bioavailability owing to poor solubility, which results in inadequate gut absorption, further reducing the therapeutic effect of these nutraceuticals.^25^, ^26^, ^27^, ^28^, ^29^ Therefore, it is essential to identify and develop systems that can protect sensitive nutraceuticals like berberine and ginkgetin from degradation and can deliver them in a bioavailable formulation to desired tissue targets enabling their beneficial effects.

Medical nanotechnology provides ideal tools to address the hurdles associated with stability, bioavailability and functionality of nutraceuticals. The development of synthetic nanoparticles made of lipid, gold, polymers and liposomes has resulted in improved bioavailability, stability, controlled and targeted release and improved functionality.^27^, ^28^, ^29^, ^30^ Despite a wide range of beneficial effects, there are health concerns related to the use of synthetic nanoparticles. Accumulating data link the use of synthetic nanoparticles to toxicity, immune incompatibility and unfavourable distribution profiles in vivo.^27^, ^28^, ^29^, ^30^, ^31^ Since the discovery that natural nanosized EVs released from mammalian cells shuttle between cells and deliver their cargo to modulate cellular function, the application of EVs as drug carriers has gained considerable interest.^32^ For an example, EVs derived from mammalian cells or milk loaded with curcumin provide protection in in vitro and in vivo disease models.^27^, ^28^, ^29^, ^30^, ^31^, ^32^ However, large‐scale production of EVs from mammalian cells for therapeutic use is challenging. Recently, it has been reported that plant cells secrete functionally active EVs similar to mammalian cell‐derived EVs.^33^, ^34^, ^35^, ^36^, ^37^, ^38^, ^39^, ^40^ EVs released from grapefruit, ginger, broccoli and lemon participate in interspecies communication.^34^ Grape, lemon and ginger‐derived EVs have been shown to have numerous beneficial activities in vitro and in vivo including induction of expression of stem cell growth genes, modulation of gut microbiota, promotion of anti‐cancer activity, suppression of inflammation, upregulation of tissue regeneration and protection of mice against colitis.^35^, ^36^, ^37^, ^38^, ^39^, ^40^ The important biological properties of plant‐derived EVs such as low toxicity, mass scalable production opportunities and better biocompatibility, makes these natural nanoparticles better, safer and more economical candidates than synthetic nanocarriers for use in treating chronic inflammatory diseases including atherosclerosis.

The unique nutraceutical profile of avocado (*Persea Americana*, Lauraceae) makes it a nutrient rich healthy food. It is the only fruit that contains heart healthy monounsaturated fatty acids and is rich in antioxidants, polyphenols, vitamins and minerals. Consumption of avocados exerts antioxidant and anti‐inflammatory effects and has been associated with decreased risk of inflammatory diseases including cardiovascular disease.^39^, ^40^ Here, we show that the pulp of avocado contains large numbers of EVs. We have characterized their biophysical properties using transmission electron microscopy and atomic force microscopy. We found that avocado‐derived EVs were rapidly taken up by peritoneal macrophages in a time‐ and dose‐dependent manner. Using a freeze thaw method, we showed that avocado‐derived EVs could be efficiently loaded with ginkgetin and berberine, suggesting a potential use of these natural EVs as a drug/nutraceutical carrier. Moreover, we showed that combined ginkgetin and berberine‐loaded EVs attenuated inflammation and foam cell formation in macrophages in vitro. The results of this study suggest that natural EVs can provide an effective nanocarrier for poorly soluble nutraceuticals that will be useful in the prevention of inflammation and macrophage foam cell formation.

## 2 MATERIALS AND METHODS

### 2.1 Reagents

Ginkgetin, berberine, 1,1′‐Dioctadecyl‐3,3,3′,3′‐tetramethylindocarbocyanine perchlorate (DiI) and lipopolysaccharide (LPS) from *E. coli* were purchased from Sigma‐Aldrich (St. Louis, MO) and dissolved in DMSO as stock solutions. RNeasy micro kit and RNase‐free DNase were obtained from Qiagen (Germantown, MD). Primers for TNFα, CCL2, IL6 and CD36, and iTaq Universal SYBR Green RT‐qPCR kit were obtained from Bio‐Rad (Hercules, CA). Thioglycollate was purchased from Beckton‐Dickinson (Franklin Lakes, NJ). Exosome precipitating reagent ExoQuick‐TC and Fluorocet kit were from System Biosciences (Palo Alto, CA). Phosphate buffered saline (PBS), Dulbecco's Modified Eagle Medium (DMEM), heat‐inactivated foetal bovine serum (FBS), native low‐density lipoprotein (LDL), bovine serum albumin (BSA) and cell culture related reagents were purchased from Thermo Fisher Scientific (Waltham, MA). All other chemicals were purchased from Sigma‐Aldrich or Thermo Fisher Scientific.

### 2.2 Animals and mouse peritoneal macrophages isolation

C57BL/6 mice were purchased from the Charles River Laboratories (Wilmington, MA). All experimental procedures and animal care were performed in accordance with the Institutional Animal Care and Use Committee (IACUC) guidelines and were approved by the University of Maryland‐College Park Review Committee (protocol# R‐OCT‐22‐47). Mice were housed under pathogen‐free conditions with controlled temperature and humidity, and with food and water available ad libitum. After 4 days of daily thioglycolate injections (i.p.), mice (two males and two females; aged 6–8 weeks, and average body weight of 24 g) were euthanized, and sterile ice‐cold PBS was injected into the cavity of each mouse by peritoneal lavage. Animals were euthanized by CO_2_ asphyxiation as per our IACUC guidelines. Peritoneal macrophages were carefully collected and centrifuged at 1000 *g* for 5 min. Cells were resuspended in DMEM supplemented with FBS and antibiotic‐antimycotic (Thermo Fisher Scientific) and incubated at 37°C with 5% CO_2_, as previously described.^9^, ^41^

### 2.3 Isolation of fruit‐derived EVs

EVs from four different fruit (avocado, kiwi, orange and plum) were harvested, as previously described.^33^, ^34^, ^35^, ^36^, ^37^, ^38^, ^39^, ^40^ Briefly, the pulp of each fruit was ground in sterile PBS using a glass homogenizer, followed by filtration (70 μm) to remove any large fibres. Samples were centrifuged in a fixed rotor 5415R centrifuge (Eppendorf, Hamburg, Germany) at 16000 *g* for 20 min to remove debris. A total 90 mL of supernatant was concentrated to 60 mL by centrifugation at 3700 *g* for 30 min using Amicon Ultra‐15 10K MWCO Centrifugal Filter Units (Millipore, Burlington, MA) in a swinging bucket rotor on a SORVALL Legend RT centrifuge (Thermo Fisher Scientific). To precipitate EVs, ExoQuick‐TC reagent was added to the sample (1:10) and refrigerated overnight. The sample was centrifuged twice at 2000 *g* for 25 and 5 min, respectively, and the supernatant was aspirated. The pellet containing purified EVs was filtered through a 0.22 μm membrane followed by resuspension in PBS for direct use in subsequent studies. For the quantification of protein concentration, EVs in PBS were lysed by the addition of an equal volume of RIPA buffer (Thermo Fisher Scientific) containing protease‐phosphatase Inhibitor Cocktail (Thermo Fisher Scientific). EVs were quantified by measuring protein concentration using a bicinchoninic acid (BCA) protein assay kit (Thermo Fisher Scientific). To quantify the number of EVs isolated from four different fruit, we used a FluoroCet Exosome Quantitation Kit according to the manufacturer's instructions (System Biosciences).

### 2.4 Transmission electron microscopy

Fruit‐derived EVs were visualized using transmission electron microscopy.^34^, ^35^, ^36^, ^37^ Briefly, the purified EVs were further diluted in water and 5 μL of sample was loaded onto a 200‐mesh Formvar/carbon coated copper grid (Electron Microscopy Sciences, Hatfield, PA). Grids were washed with distilled water and dried on filter paper. For negative staining, 1% uranyl acetate was added on the grid for 10 sec and remaining solution was wicked off with filter paper. Grids were then examined on a JEOL 100CXII transmission electron microscope at 80 kV.

### 2.5 Atomic force microscopy

The topography of EVs was determined by a JPK Nanowizard 4 AFM (Bruker Nano GmbH, Berlin, Germany). The AFM, mounted on an inverted optical microscope (Nikon Eclipse TE200, Melville, NY, USA) was used to visualize AFM tip. EVs from avocado (PDN^Avo^) were deposited on a poly‐L‐ornithine‐coated (Sigma), freshly cleaved, 15 mm AFM grade mica sheet (TedPella, USA). The quantitative imaging (QI) of EVs was carried out in PBS at 30°C, by acquiring QI maps of 128 × 128 pixels. For QI measurements, we used AC‐40 cantilevers (Bruker, Camarillo, CA, USA) with a nominal resonance frequency of 25 kHz in water, spring constant of 0.09 N/m, gold/chromium coating on the detector side and a tetrahedral silicon probe with 8 nm radius. The sensitivity and spring constant of each cantilever was individually calibrated before the measurements. Image processing was performed using JPK data processing software.^42^, ^43^, ^44^, ^45^

### 2.6 Physicochemical characterization of EVs

EVs were obtained in pellet form and then dissociated in 1× PBS using a glass homogenizer. Their total protein content was determined using the BCA assay (Pierce™ BCA Protein Assay Kit; Thermo Scientific, USA), following the manufacturer's instructions. These EVs were then diluted in 1× PBS to achieve a concentration of 1 mg/mL. Their size, polydispersity and zeta potential were studied using the NanoBrook Omni (Brookhaven Instruments Corporation, NY, USA). For particle size distribution and polydispersity analysis, the EVs were diluted 100 times in 1× PBS and then filtered using a 0.4 μ syringe to prevent further aggregation. Measurements were taken with dynamic light scattering (DLS) at conditions of 25°C, a 90‐degree scattering angle and a 640 nm wavelength. To measure zeta potential, the EVs were again diluted 100 times in 1× PBS. Zeta Phase Analysis Light Scattering (ZetaPALS) was employed at 25°C, with a cycle run of 20 and a measurement 3 out of 3 sample sets. Data interpretation was based on the Smoluchowski model.

### 2.7 ExoGlow labeling of avocado‐derived EVs and their uptake by mouse peritoneal macrophages

To monitor the uptake of EV^Avo^ by peritoneal macrophages, EV^Avo^ was labelled with Exo‐GLOW Membrane EV Labeling Kit (System Biosciences) per the manufacturer's instructions.^46^ Briefly, EV^Avo^ (300 μg) was mixed with the Exoglow fluorescent dye and incubated at RT for 30 min in the dark. We used 10 K MWCO buffer exchange tubes and centrifuged samples at 12000 *g* for 10 min to remove free unlabeled dye. Labelled EV^Avo^ (2 or 10 μg/mL) was then added to macrophages and their uptake by the cells at different time points was visualized by fluorescence microscopy (Zeiss Axio Observer, White Plains, NY).

### 2.8 Immunoblots

Thioglycolate‐elicited peritoneal macrophages were placed in DMEM with 10% FBS and allowed to grow for 48 h. After this period, cells that didn't adhere were removed, and the plate was supplemented with DMEM containing 1% BSA. This was followed by a 3‐h incubation. Post‐incubation, the cells underwent treatment with various formulations: empty avocado extracellular vesicles (EV^Avo^), berberine‐loaded EV (EV^Avo(B)^), ginkgetin‐loaded EV (EV^Avo(G)^) and a combination of GGT‐BBR in EV (EV^Avo(B+G)^). The standard was set to 1 mg EV for every 1 × 10^6^ cells. This treatment lasted 15 min in conditions of 37°C and an atmosphere of 5% CO2. Subsequently, some cells were exposed to LPS (100 ng/mL; Sigma Aldrich Cat# L5418‐2ML) for a duration of 60 min. After treatments, cells were harvested for lysate using the RIPA lysis buffer (Thermo Cat#89900) containing both protease and phosphatase inhibitors (Thermo Cat#78430). The aim was to gauge the expression levels of phosphorylated‐NFκB p65 (Cell signalling technology, cat#93H1), total‐NFκB p65 (Cell signalling technology, cat#D14E12), NLRP3 (Cell signalling technology, cat#D4D8T) and Actin proteins. For loading control purposes, blots were reintroduced to anti‐Actin IgG (Cell signalling technology, cat#4970).

### 2.9 Loading of ginkgetin and berberine into avocado‐derived EV^Avo^

The loading of ginkgetin (GGT or G) and berberine (BBR or B) into EV^Avo^ was achieved by three freeze–thaw cycles as described.^31^, ^32^ Equal amounts of GGT and BBR (dissolved in DMSO) were mixed and incubated with EV^Avo^ (in PBS) at room temperature (RT) for 15 min. The mixture was then frozen in liquid nitrogen for 1 min followed by thawing at RT. The EV^Avo^ and nutraceuticals formulation was then transferred to 10 K MWCO centrifugal filter units (Thermo Fisher Scientific), centrifuged at 14000 *g* for 10 min and resuspended in PBS to remove free nutraceuticals. The concentration of free GGT and BBR was calculated from a standard curve prepared by measuring the fluorescence intensity of known concentrations of free GGT at 330 nm and that of free BBR at 420 nm (FLUOstar Optima, BMG Labtech, Cary, NC). The GGT and BBR encapsulated in EV^Avo^ was expressed as loading efficiency calculated as previously reported.^31^, ^32^

### 2.10 Real‐time quantitative RT‐PCR (qRT‐PCR)

Mouse peritoneal macrophages were treated with a fixed concentration of EV^Avo^ loaded with GGT and BBR (1 μg EV^Avo^ to 50 cells), followed by incubation with 100 ng/mL *E. Coli*‐derived LPS for 12 h at 37°C in DMEM supplemented with 1% BSA. Twelve hours post‐incubation, cells were harvested, and total mRNA was extracted from mouse peritoneal macrophages using the RNeasy micro kit per manufacturer's instructions (Qiagen). Isolated mRNA was treated with RNase‐Free DNase and cleaned using a kit. The concentration and purity of the RNA were measured in a Nanodrop‐2000 spectrophotometer (Thermo Fisher Scientific). The cDNA synthesis and PCR amplification was performed at 95–60°C for 40 cycles in a CFX96 cycler (Bio‐Rad) using iTaq Universal SYBR Green One‐Step Kit and Bio‐rad PrimePCR mouse specific primers for *Tnfα* (qMmuCED0004141), *Il1* (qMmuCID0005641), *Cd36* (qMmuCID0014852) and *Il6* (qMmuCID0005613). The fluorescence cyclic threshold (Ct) values for genes of interest were normalized to the average Ct for *Gapdh* (qMmuCID0018612), and the fold change of target gene expression was calculated using the Ct value.

### 2.11 Foam cell formation

Macrophage‐derived foam cells were identified by Oil‐red‐O staining.^9^, ^41^ The oxidized LDL (oxLDL) was prepared by incubating native LDL with copper sulphate (5 μM) for 12 h at 37°C, protected from light, followed by dialysis against PBS to remove excess copper sulphate.^9^, ^41^ The peritoneal macrophages from C57BL/6 mice were pre‐treated with GGT and BBR loaded EV^Avo^ at ratios of 1:1000, 1:250 and 1:50 (EV^Avo^ μg/cell number) for 15 min, followed by incubation with 50 μg/mL oxLDL for 12 h at 37°C in DMEM supplied with 10% FBS. Cells were fixed with ice‐cold 10% phosphate buffered formalin for 10 min and stained for 1 min with freshly prepared filtered Oil‐Red‐O solution followed by three rinses with PBS. Several fields were randomly selected and analysed for foam cells using a Zeiss Axio Observer (20×) microscope and NIH ImageJ software.

### 2.12 DiI‐labelled oxLDL binding and uptake by mouse peritoneal macrophages

To prepare DiI‐labelled oxLDL (oxLDL‐DiI), DiI (2 mg/mL) was mixed with oxLDL (2 mg/mL) in a 1:3 ratio and incubated for 24 h at 37°C in the dark. The solution was diluted with sterile PBS, transferred to the upper chamber of a 10 K MWCO centrifugal tube and centrifuged at 12000 *g* for 10 min to remove free dye. Peritoneal macrophages were seeded on an 8‐well chamber slide, incubated for 24 h and pre‐treated with GGT and BBR loaded EV^Avo^ at a 1:250 and a 1:50 ratio (μg EV^Avo^/cell number) for 1 h at 37°C and 5% CO_2_. One hour after the treatment with EV^Avo^, cells were incubated with oxLDL‐DiI (5 μg/mL) for 1 h at 4°C, washed with PBS, fixed and binding was assessed.^9^ For the uptake assay,^9^ cells were incubated with oxLDL‐DiI (5 μg/mL) for 30 min at 37°C in DMEM containing 10% FBS, washed with PBS and fixed. Fluorescence intensity of DiI‐labelled oxLDL per cell was examined on a Zeiss Axio Observer (20×) microscope and quantified using NIH ImageJ software.

### 2.13 Data analysis

Statistical analyses were performed using Student's *t*‐test and one‐way analysis of variance, using GraphPad Prism (GraphPad Prism Software, La Jolla, CA). Experiments were repeated 3 times and values are expressed as mean ± SEM. A ‘*p*’ value of <0.05 was considered to indicate statistical significance.

## 3 RESULTS

### 3.1 Isolation and characterization of plant‐derived EVs

**FIGURE 1:**
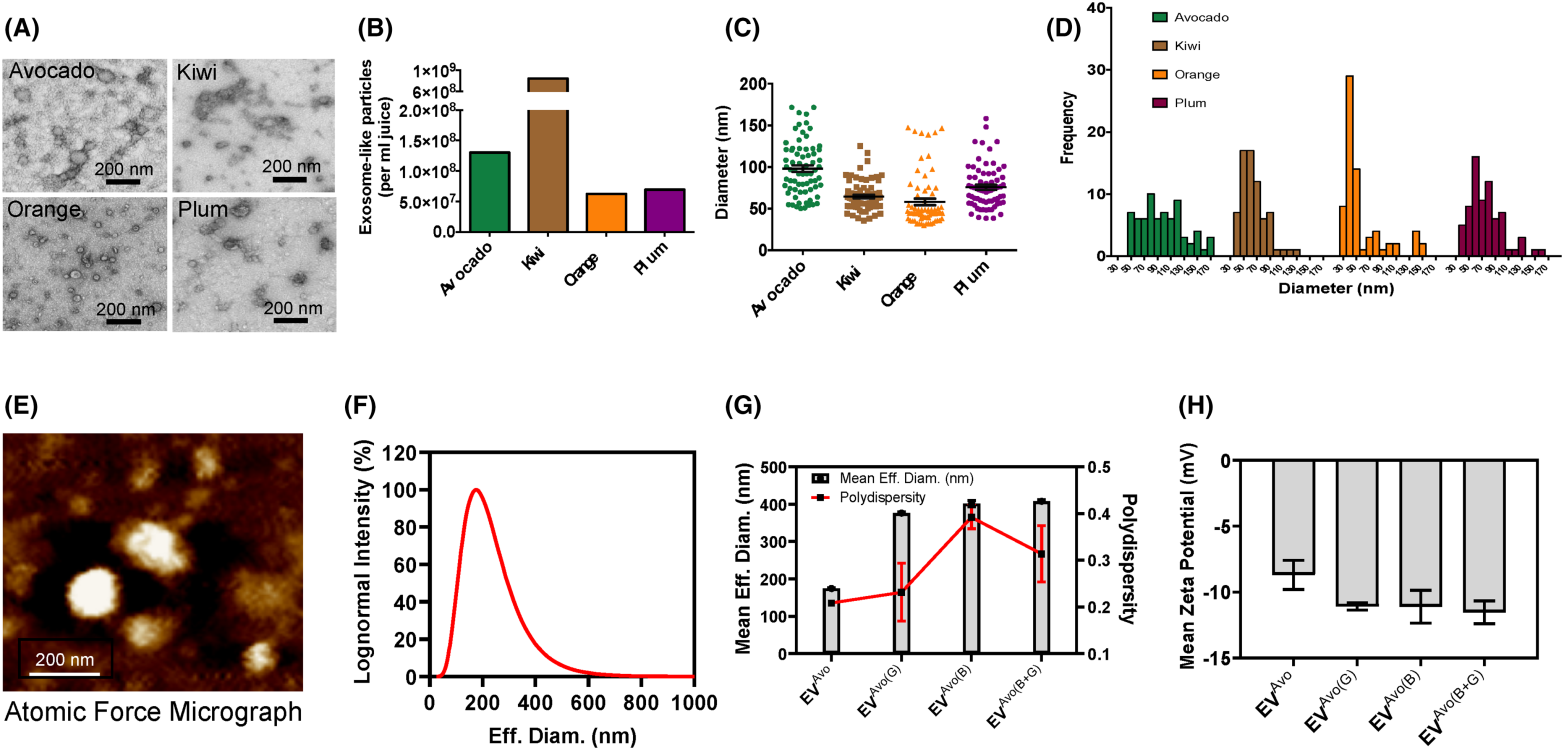
Isolation and characterization of extracellular vesicles (EVs) from avocado, kiwi, orange and plum. (A) Transmission electron micrographs (negative staining with uranyl acetate) of EVs show a typical spherical cup‐shaped morphology and size ranging from 30 to 170 nm. (B) Bar graph shows the number of EVs isolated from different fruits. (C) Bar graph shows EV size distribution profile, and (D) frequency of different sized EV population from different fruits. (E) Representative atomic force micrograph of EVs harvested from avocado. Bar graph and plot profile shows EV size distribution, polydispersity index (F and G) and zeta potential (H) as evaluated by dynamic light scattering analysis.

We used ultrafiltration followed by a polymer‐based precipitation method for the isolation of plant‐derived EVs from the pulps of avocado, plum, kiwi and orange.^35^, ^38^, ^47^, ^48^ Isolated EVs were characterized by transmission electron microscopic (TEM) analysis focusing on two criteria, spherical morphology and size, which ranged from 30 to 170 nm, characteristic of EVs in a dry state.^35^, ^38^, ^47^, ^48^ TEM images (Figure 1A) show that the EVs from avocado, plum, kiwi and orange exhibited a morphological ultrastructure and size similar to mammalian EVs.^48^ We found that all tested fruits contained abundant EVs, with plum and orange having the minimum number (Figure 1B). The EVs were nanosized and distributed within the expected size range for plant‐derived EVs (30–170 nm) (Figure 1C,D), consistent with previous studies.^35^, ^38^, ^47^, ^48^ Among all the fruits, the size of the EV population having an average diameter of 100 nm was maximal in avocado EVs (Figure 1A,C,D). The maximal EV populations in the other fruit were 30–50 nm in oranges, 50–70 nm in kiwis and 60–80 nm in plums (Figure 1D). However, there was homogeneity in the distribution of 50–120 nm diameter EVs isolated from avocado, which made it a preferred vehicle for loading nutraceutical agents. Atomic force microscopy (AFM) analysis confirmed that EVs from avocado were spherical in shape, and the diameter of individual EV was around 100 nm, which is consistent with the values obtained from TEM (Figure 1E). Additional data from dynamic light scattering analysis using NanoBrook Omni instrument further characterized avocado‐EV by analysing their size, polydispersity index and zeta potential (Figure 1F–H). Together, these results suggest that avocado contains large population of EVs.

### 3.2 Avocado‐derived EVs as carriers of nutraceutical agents

**FIGURE 2:**
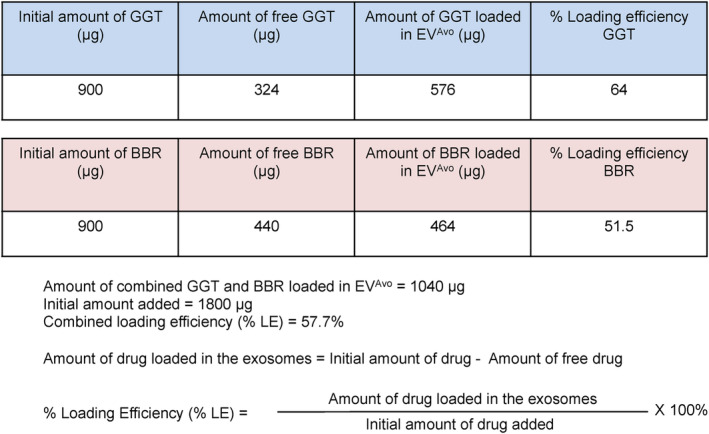
Successful loading of ginkgetin and berberine in EVs isolated from avocado. Equal amounts of ginkgetin (GGT) and berberine (BBR) were mixed and loaded in EVs isolated from avocado (EV^Avo^) by the freeze–thaw method. Loading efficiency of BBR and GGT in EV^Avo^ was calculated from the initial amount of nutraceuticals added and the amount of residual free nutraceuticals after loading.

To utilize EVs from avocado (EV^Avo^) as a carrier of nutraceuticals, a combination of incubation and rapid freeze–thaw cycles were employed to load nutraceuticals in EV^Avo^. EV^Avo^ was incubated with nutraceutical compounds ginkgetin (GGT) and berberine (BBR) for 15 min at room temperature followed by three cycles of rapid freeze–thaw to allow diffusion of compounds into EV^Avo^. Following incubation, the mixture was frozen in liquid nitrogen for 1 min, thawed at room temperature and then subjected to ultrafiltration to remove free compounds. This resulted in loading of 64% of the GGT and 51.5% of the BBR onto the EV^Avo^, providing a combined nutraceutical loading efficiency of 57.7% (Figure 2). The final total loading was 1.04 mg of GGT and BBR to 1.8 mg of EV^Avo^, resulting in loading of 57.7%.

### 3.3 Cellular uptake of avocado‐derived EVs

**FIGURE 3:**
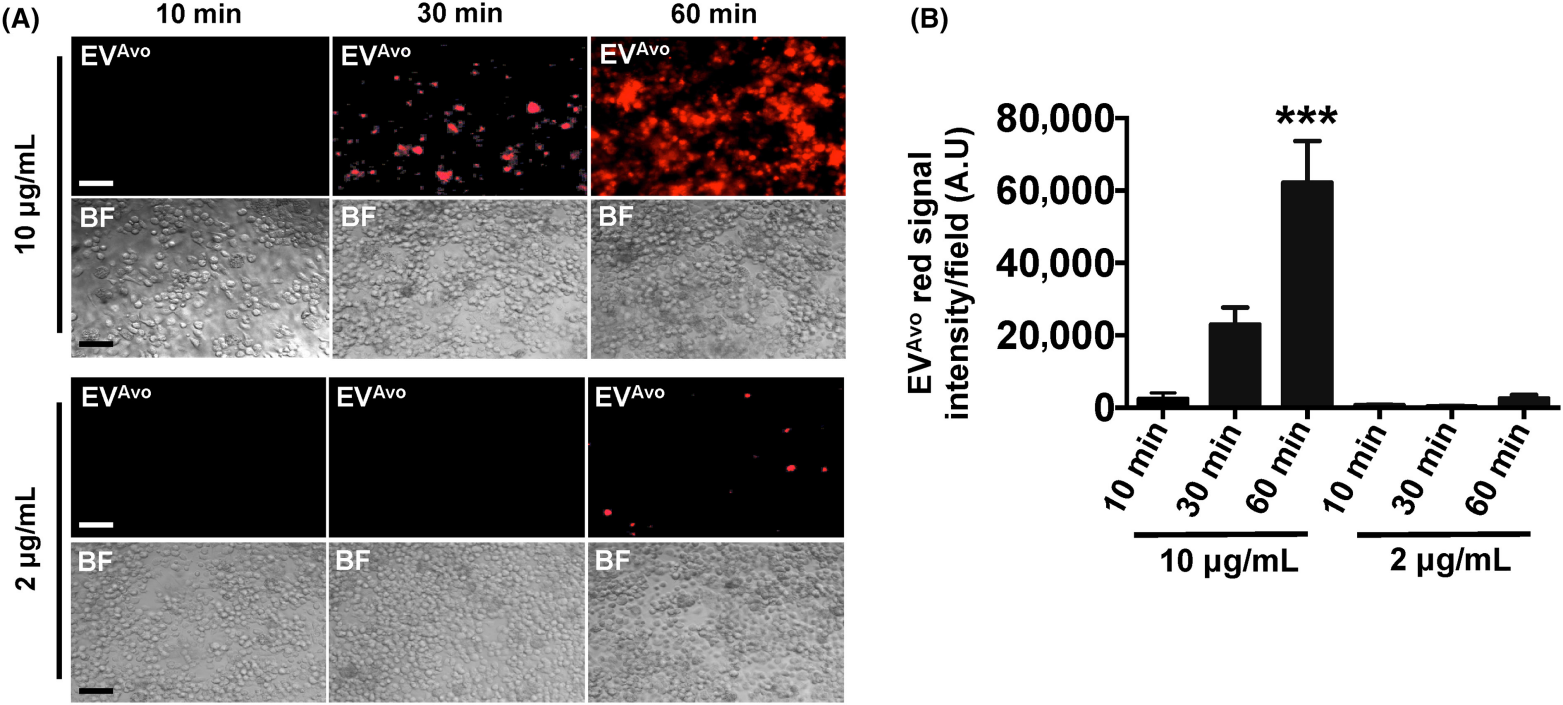
EV^Avo^ are taken up by macrophages. (A) Fluorescence microscopy and bright field (BF) images showing temporal and dose‐dependent uptake of fluorescent dye‐labelled EV^Avo^ by mouse peritoneal macrophages. (B) Bar graph shows quantification of results from A. Data are presented as the mean ± SEM; ***p < 0.001, *t*‐test, Scale bar, 20 μm.

To determine whether EV^Avo^ are taken up by mouse peritoneal macrophages, EV^Avo^ were labelled with lipophilic dye from the ExoGlow‐Membrane EV Labeling Kit, and the labelled EV^Avo^ were incubated with peritoneal macrophages. Macrophages incubated with 10 μg/mL of EV^Avo^ for 30 min or 60 min internalized EV^Avo^ in a time‐dependent manner (Figure 3). Within 30 min of incubation, the labelled EV^Avo^ were detected in macrophages, indicating that the EV^Avo^ were quickly taken up by macrophages. After 60 min incubation, approximately 80% of the cells had internalized EV^Avo^. However, when macrophages were incubated with 2 μg/mL EV^Avo^ uptake occurred at a reduced rate. These results indicate that uptake of EV^Avo^ by peritoneal macrophages occurred rapidly in a time‐ and dose‐dependent manner.

### 3.4 Nutraceutical‐loaded EV^Avo^ downregulates the expression of inflammatory genes

**FIGURE 4:**
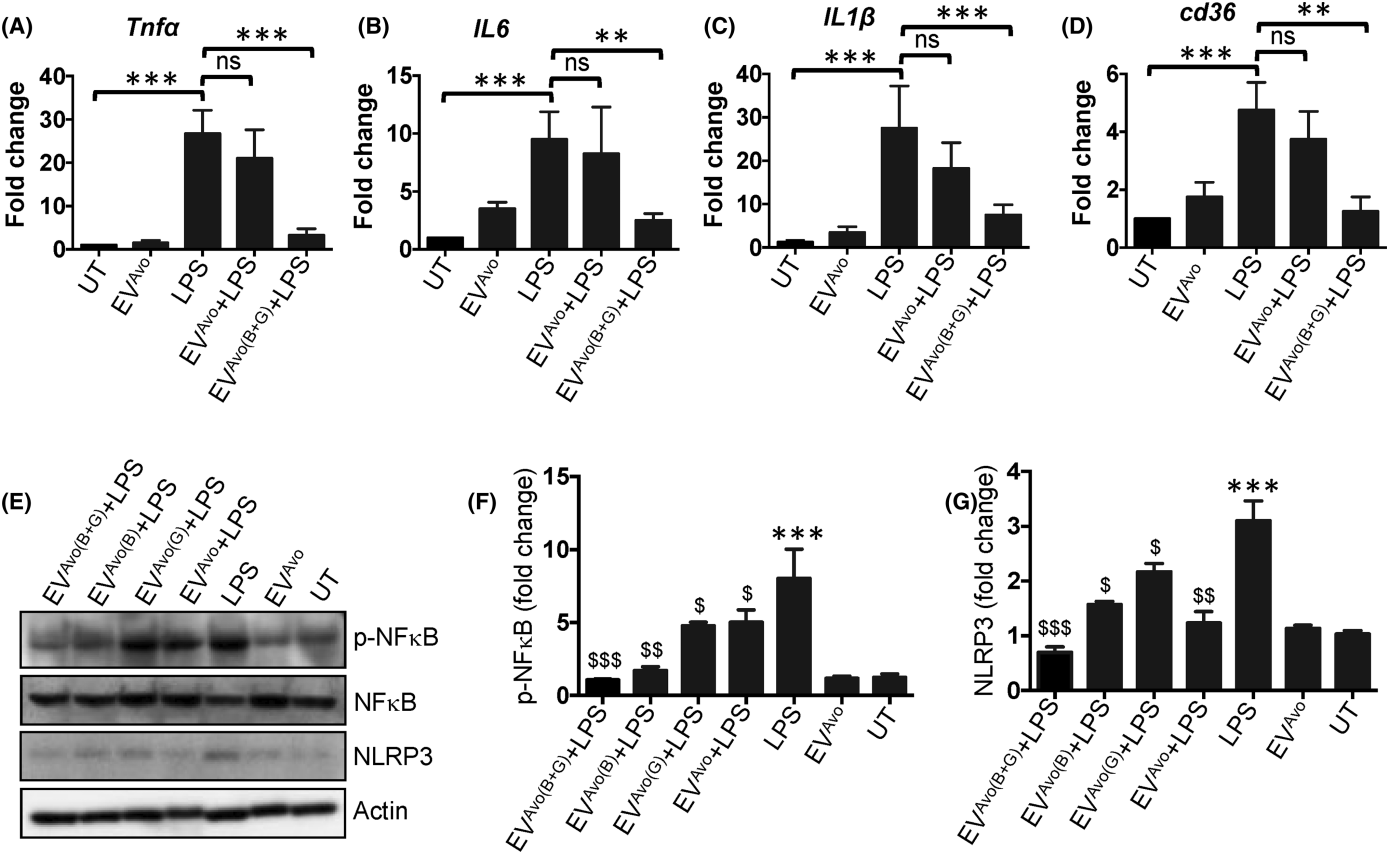
Suppression of inflammatory gene expression by berberine and ginkgetin‐loaded EV (EV^Avo(B+G)^). Peritoneal macrophages were incubated with EV^Avo(B+G)^ plus 100 ng/mL of *E. coli* LPS for 12 h. Real‐time quantitative PCR detection of mRNA expression of *Tnfα* (A), *Il6* (B), *Il1β* (C) and *Cd36* (D) genes. Untreated (UT) macrophages were used as a negative control for LPS stimulation. *Gapdh* was used as a housekeeping gene for normalization of the data. Data are presented as the mean ± SEM; **p < 0.01, ***p < 0.001, *t*‐test. (E) Representative immunoblot images showing levels of p‐NFκB p65, NFκB p65, NLRP3 and Actin proteins (loading control). (F and G) Quantitative analysis of p‐NFκB p65 and NLRP3 levels after normalizing with total NFκB p65 and Actin protein expression respectively. Student's *t*‐test; ***p < 0.001 vs untreated (UT) condition; ^$^ *p* < 0.05, ^$$^ *p* < 0.01, ^$$$^ *p* < 0.001 vs LPS treated condition; *t*‐test, mean ± SEM.

To determine the anti‐inflammatory effects of EV^Avo^ loaded with GGT and BBR (EV^Avo(B+G)^), macrophages were incubated with or without GGT/BBR loaded EV^Avo^ in the presence of *E. coli* LPS. As expected, LPS stimulation caused an increase in the expression of *Tnfα*, *Il6, Il1β* and *Cd36* genes (Figure 4A–D). Comparatively, the expression levels of these genes were reduced in EV^Avo(B+G)^‐treated macrophages compared to their expression in LPS or LPS plus EV^Avo^ treated macrophages (Figure 4A–D). Our additional in vitro data show that EV^Avo^, which are loaded with a mixture of ginkgetin and berberine (referred to as EV^Avo(B+G)^) display elevated anti‐inflammatory potentials compared to EV^Avo^ loaded exclusively with either ginkgetin or berberine. This augmentation is substantiated by the suppression of activation of NFκB and NLRP3 (Figure 4E–G). These results suggest that EV^Avo(B+G)^ might possess synergistic qualities favourable for mitigating inflammatory responses in macrophages. Collectively, our results indicate that treatment with GGT/BBR loaded EV^Avo^ (EV^Avo(B+G)^) downregulates the expression of inflammation‐related genes in mouse peritoneal macrophages challenged with LPS.

### 3.5 Nutraceutical‐loaded EV^Avo^ suppresses oxLDL‐induced macrophage foam cell formation

**FIGURE 5:**
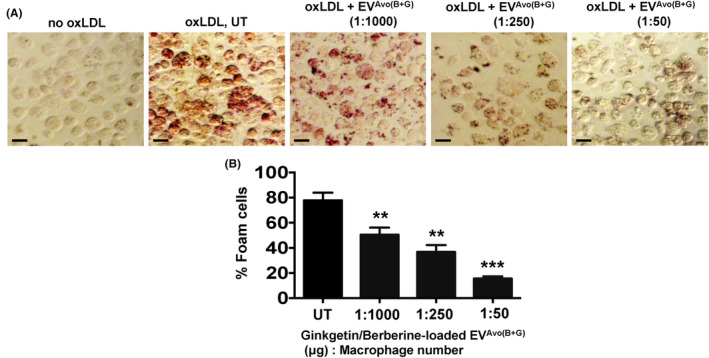
Ginkgetin and berberine‐loaded EV^Avo^ attenuate macrophage foam cell formation. Mouse peritoneal macrophages were treated with EV^Avo(B+G)^ (1 μg EV per number of macrophages treated: 1:50, 1:250, 1:1000) and oxLDL (50 μg/mL) for 12 h. (A) Representative images showing Oil‐Red‐O staining of foam cells. (B) Quantitation of results from five different fields per condition shown in A. Number of foam cells is expressed as percentage of total cells counted in random fields. Data are presented as the mean ± SEM; **p < 0.01 and ***p < 0.001 vs oxLDL treated control (UT); *n* = 20 cells/field, *t*‐test, Scale bar, 20 μm.

Atherosclerosis, a chronic inflammatory disease, is linked to increased expression of macrophage CD36, a receptor for oxLDL.^4^, ^5^, ^6^, ^7^, ^8^, ^9^ Since we found that EV^Avo(B+G)^ treatment suppressed expression of *Cd36 mRNA* in macrophages following LPS challenge, we next investigated whether treatment with EV^Avo(B+G)^ abrogated oxLDL‐induced foam cell formation in peritoneal macrophages. As expected, we found that oxLDL‐induced foam cell formation in macrophages (Figure 5A,B). As the EV^Avo(B+G)^ concentration (nanoparticle μg to cell number) was increased, there was a decrease in the percentage of macrophage‐derived foam cells (Figure 5B). These results indicate that treatment with EV^Avo(B+G)^ inhibits oxLDL‐induced macrophage foam cell formation.

### 3.6 Nutraceutical‐loaded EV^Avo^ inhibits the uptake of oxLDL, but not its binding to macrophages

**FIGURE 6:**
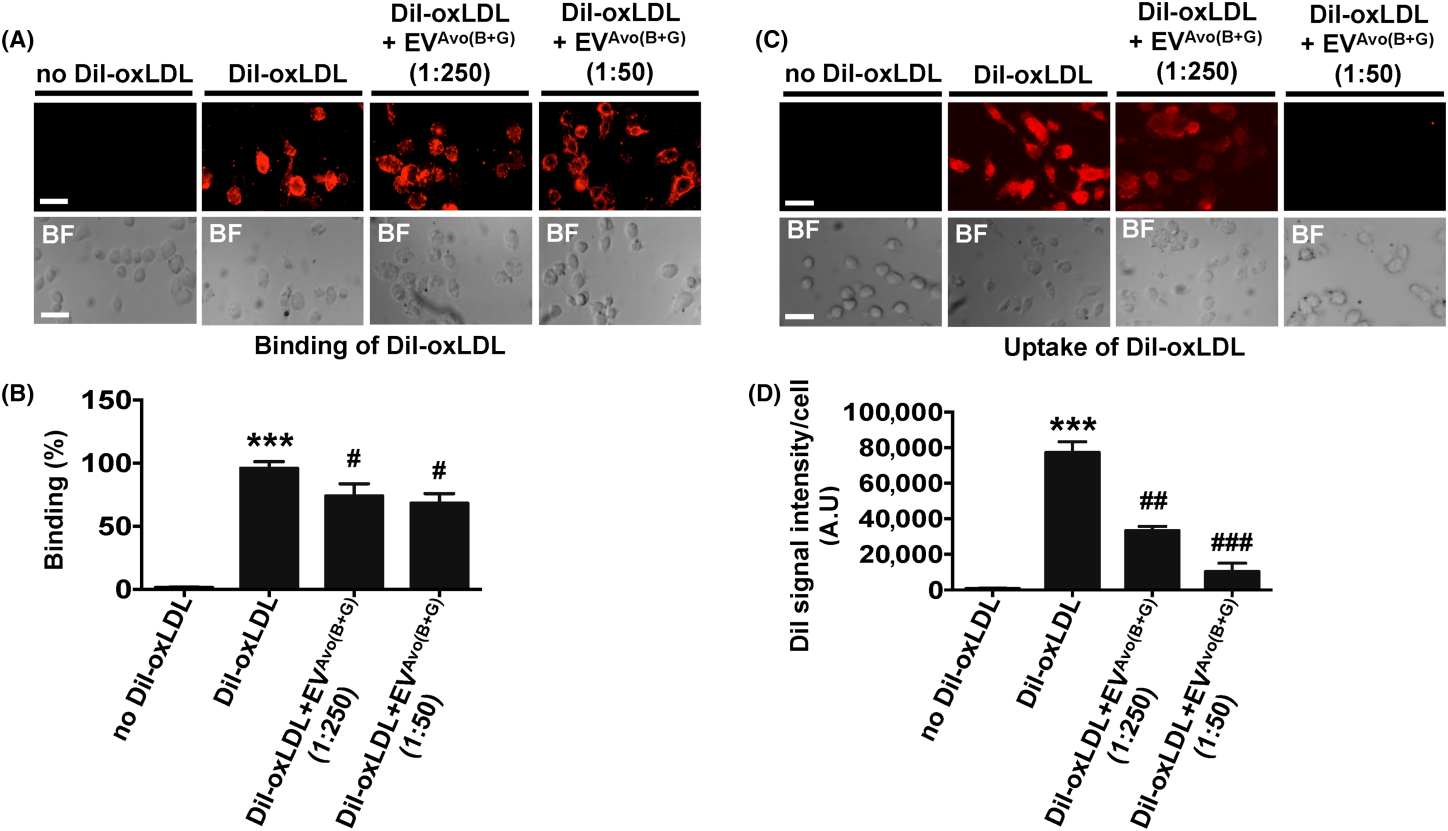
Ginkgetin and berberine‐loaded EV^Avo^ inhibit oxLDL uptake by macrophages but not cell surface binding. (A) Cells were pre‐treated with EV^Avo(B+G)^ (1 μg EV per number of macrophages treated: 1:50, 1:250) for 1 h at 37°C. Cells were then incubated with or without DiI‐labelled oxLDL (DiI‐oxLDL; 5 μg/mL) for 1 h at 4°C to assess oxLDL binding. Representative images are shown for DiI‐oxLDL binding on the cell surface (original magnification, 20×). (B) Quantification of results from A. Data are presented as the mean ± SEM; ***p < 0.001 vs without DiI‐oxLDL and ^#^ *p* < 0.05 vs DiI‐oxLDL control; *n* = 20 cells/field. (C) Cells were pre‐treated with EV^Avo(B+G)^ as in A. Cells were then incubated with or without DiI‐oxLDL (5 μg/mL) for 30 min at 37°C, and oxLDL uptake was assessed. (D) Quantification of results from C. Bar graphs show mean DiI fluorescence intensity. Data are presented as the mean ± SEM; ***p < 0.001 vs without DiI‐oxLDL treatment, ^##^ *p* < 0.001 and ^###^ *p* < 0.001 vs DiI‐oxLDL; *n* = 20 cells/field, *t*‐test. A.U, arbitrary units; Scale bar, 20 μm.

CD36 plays a major role in binding and uptake of oxLDL during lipid‐laden foam cell formation, a critical process in atherosclerosis.^4^, ^5^, ^6^, ^7^, ^8^, ^9^ We sought to determine whether the inhibitory effect of EV^Avo(B+G)^ on oxLDL‐induced macrophage foam cell formation was caused by its effect on binding and/or uptake of oxLDL in macrophages. Our results show that treatment with EV^Avo(B+G)^ did not influence the binding of fluorescence dye‐labed oxLDL (DiI‐oxLDL) to macrophages, even at higher concentration (1:50) (Figure 6A,B). In contrast, we found that treatment with EV^Avo(B+G)^ reduced uptake of DiI‐oxLDL in macrophages (Figure 6C,D). Taken together, these findings suggest that uptake of oxLDL by macrophages treated with EV^Avo(B+G)^ is reduced.

## 4 DISCUSSION

Although currently approved pharmacotherapies for CVD are effective in reducing mortality, a large percentage of patients receiving current pharmacotherapies still harbour a discernible residual risk of a CVD‐linked complication, and may develop unwanted side effects such as muscle pain and hepatic toxicity.^1^, ^2^, ^3^, ^14^, ^15^, ^16^ Therefore, there is an urgent need to develop alternative, safe and targeted therapeutics for atherosclerosis that can be administered alone or in combination with current pharmacotherapies to limit inflammation and other pro‐atherogenic responses in atherosclerosis. One potential chemopreventive avenue for prevention of atherosclerosis is development of natural product‐derived nutraceuticals/bioactives that have anti‐inflammatory properties.^16^ For example, berberine (from *Berberis aristata*), an alkaloid and a bacteriostatic agent and ginkgetin (from *G. biloba)*, a biflavonoid, are known to have anti‐oxidative and anti‐inflammatory effects and have been shown to be beneficial against various inflammatory/oxidative conditions in vitro and in vivo.^18^, ^19^, ^20^, ^21^, ^22^, ^23^ However, both berberine and ginkgetin have low stability, poor penetration of target tissues and cells and low bioavailability, which reduces the chemotherapeutic benefit of these bioactives.^25^, ^26^, ^27^, ^28^, ^29^ Thus, identifying and developing a system that protects sensitive nutraceuticals such as berberine and ginkgetin from degradation while delivering them to desired tissues and cells to exert their maximal chemopreventive effects is highly warranted.

Nanotechnologies provide ideal tools for addressing the hurdles associated with stability, bioavailability and functionality of nutraceuticals. Since the discovery that natural nanosized EVs released from mammalian cells shuttle between cells, and deliver their cargo to modulate cellular function, the application of exosomes as drug and bioactive carriers has gained considerable interest.^27^, ^28^, ^29^, ^30^, ^31^, ^32^ However, large‐scale production of EVs from mammalian cells for therapeutic/chemopreventive use is challenging. Recently, it was reported that plant cells secrete functionally active nanosized particles similar to mammalian cell‐derived EVs.^33^, ^34^, ^35^, ^36^, ^37^, ^38^, ^39^, ^40^ Grape, lemon and ginger‐derived edible EVs were shown to have numerous beneficial activities in vitro and in vivo, including induction of the expression of stem cell growth genes, modulation of gut microbiota and suppression of inflammation.^35^, ^36^, ^37^, ^38^, ^39^, ^40^ Furthermore, orally administered plant‐derived EVs were shown to be resistant to degradation by saliva and by the acidic environment of the stomach.^34^, ^35^, ^36^ Therefore, using plant‐derived EVs as a vehicle for delivery of bioactives might provide health benefits in people suffering from chronic diseases such as atherosclerosis.

Consumption of avocados provides antioxidant and anti‐inflammatory effects, which has been associated with decreased risk of inflammatory diseases including cardiovascular disease.^39^, ^40^ Herein, we have shown that the pulp of avocado contains large numbers of EVs, and we have characterized their biophysical properties (size, polydispersity and zeta potential) using transmission electron microscopy, dynamic light scattering analysis and atomic force microscopy. We found that avocado‐derived EVs were rapidly taken up by peritoneal macrophages. Interestingly, our findings indicate that the uptake of EV^Avo^ into macrophages occurs in a manner dependent on both dose and time, a phenomenon that aligns with the reported uptake of EV derived from cardiac progenitor cells in cardiomyocytes.^49^ Using a freeze thaw method, we found that avocado‐derived EVs could be efficiently loaded with ginkgetin and berberine, suggesting a potential use of these natural EVs as bioactive carriers. Furthermore, the results we found demonstrates that EV derived from avocados (EV^Avo^) imbued with a blend of ginkgetin and berberine, denoted as EV^Avo(B+G)^, manifest enhanced anti‐inflammatory capabilities relative to EV^Avo^ loaded solely with either ginkgetin or berberine in vitro. This enhancement is evidenced by the reduction in the expression of genes related to inflammation, the attenuation of the activation of NFκB and NLRP3 and the inhibition of foam cell production in macrophages. These novel findings suggest that EV^Avo(B+G)^ may harbour synergistic properties beneficial in ameliorating inflammatory reactions within macrophages.

Our mechanistic studies show that EV^Avo(B+G)^ inhibits oxLDL internalization but not its cell surface binding during macrophage foam cell formation. Our studies do have several limitations: (i) it remains to be determined whether EV^Avo(B+G)^ can exhibit its anti‐inflammatory/atherogenic effects in human macrophages in vitro and within an in vivo atherogenic model; (ii) there is a need for comparison of the anti‐inflammatory/atherogenic effects of EV^Avo(B+G)^ with EV derived from kiwi, orange or plum; and (iii) a precise elucidation of the molecular mechanism through which EV^Avo(B+G)^ imparts its anti‐inflammatory/atherogenic effects is requisite. In summary, our results suggest that avocado‐derived EVs can be used as effective nanocarriers for poorly soluble bioactives to mitigate inflammation and atherogenic response in macrophages. Using EV^Avo^ as a nutraceutical compound carrier may provide an improved and/or alternative strategy to suppress/mitigate atherosclerosis by limiting inflammation and other pro‐atherogenic responses.

## AUTHOR CONTRIBUTIONS

**Shweta Sharma:** Data curation (equal); formal analysis (equal); investigation (equal); writing – original draft (equal). **Manisha Mahanty:** Data curation (equal); formal analysis (equal); investigation (equal). **Suneha G. Rahaman:** Data curation (equal); formal analysis (equal); investigation (equal); writing – original draft (equal). **Pritha Mukherjee:** Data curation (equal); formal analysis (equal); investigation (equal). **Bidisha Dutta:** Conceptualization (equal); data curation (equal); formal analysis (equal); investigation (equal). **Mohammad Imran Khan:** Data curation (equal); formal analysis (equal); methodology (equal). **Karunakaran Reddy Sankaran:** Data curation (equal); formal analysis (equal); methodology (equal). **Xiaoming He:** Conceptualization (equal); formal analysis (equal); investigation (equal); methodology (equal). **Lakshmyya Kesavalu:** Formal analysis (equal); methodology (equal). **Wei Li:** Conceptualization (equal); formal analysis (equal); methodology (equal). **Shaik O. Rahaman:** Conceptualization (equal); formal analysis (equal); funding acquisition (equal); investigation (equal); project administration (equal); supervision (equal); writing – original draft (equal); writing – review and editing (equal).

## CONFLICT OF INTEREST STATEMENT

The authors declare no potential conflicts of interest as it relates to the research, authorship and/or publication of this article.

## Data Availability

All data generated or analysed during this study are included in this article.
